# Immune Response of Indian Preterm Infants to Pentavalent Vaccine Varies With Component Antigens and Gestational Age

**DOI:** 10.3389/fimmu.2021.592731

**Published:** 2021-04-23

**Authors:** Archana Kulkarni-Munje, Nandini Malshe, Sonali Palkar, Aniket Amlekar, Sanjay Lalwani, Akhilesh Chandra Mishra, Vidya Arankalle

**Affiliations:** ^1^ Department of Communicable Diseases, Interactive Research School for Health Affairs, Bharati Vidyapeeth (Deemed To Be University) University, Pune, India; ^2^ Department of Paediatrics, Bharati Vidyapeeth (Deemed To Be University) University Medical College, Pune, India

**Keywords:** preterm birth, immune response, pentavalent vaccine, recall immune responses, immunological memory

## Abstract

Childhood vaccination plays critical role in protecting infants from several dreaded diseases. Of the global 15 million preterm (PT) infants with compromised immune system born annually, India contributes to >3.5 million. Generation of adequate vaccine-induced immune response needs to be ensured of their protection. Immune response of Indian PT (n = 113) and full-term (FT, n = 80) infants to pentavalent vaccine administered as per the national recommendation was studied. Antibody titers against component antigens of pentavalent vaccine, immune cells profiling (T and B cells, monocytes and dendritic cells) and plasma cytokines were determined pre- and post-vaccination. Additionally, cell-mediated recall immune responses to pentavalent antigens were evaluated after short time antigenic exposure to infant PBMCs. Irrespective of gestational age (GA), all the infants developed adequate antibody response against tetanus, diphtheria, and protective but lower antibody levels for *Haemophilus influenzae* type-b and hepatitis B in preterm infants. Lower (~74%) protective antibody response to pertussis was independent of gestational age. PT-infants exhibited lower frequencies of CD4 T cells/dendritic cells/monocytes, increased plasma IL-10 levels and lower proliferation of central and effector memory T cells than in term-infants. Proliferative central memory response of FT-infants without anti-pertussis antibodies suggests protection from subsequent infection. Responder/non-responder PT-infants lacked immunological memory and could be infected with *Bordetella*. For hepatitis B, the recall response was gestational age-dependent and antibody status-independent. Humoral/cellular immune responses of PT-infants were dependent on the type of the immunogen. Preterm infants born before 32 weeks of gestation may need an extra dose of pentavalent vaccine for long lived robust immune response.

## Introduction

The introduction of universal immunization program for infants has drastically reduced the dreaded diseases-associated mortality and morbidity in children. However, due to poor vaccination coverage, deaths continue to occur in the developing countries. Approximately 17,000 children from around the world under the age of five succumb daily, mostly to vaccine preventable diseases (VPD) (1). Globally, an estimated 14.9 million infants are born preterm, i.e., before 37 weeks of pregnancy. The risk of infection in preterm (PT) infants increases nine-fold when compared to their full term (FT) counterparts (2–5) and correlates inversely with gestational age (6); furthermore, PT infants face an increased risk of VPDs emphasizing importance of vaccination (7, 8). It is generally recommended that the preterm infants should be vaccinated using the same schedules as those usually recommended for full-term infants with the only exception of birth dose of hepatitis B vaccine, to be administered to babies weighing > 2000 g (9, 10). In view of premature immunity and introduction of more and more immunogens at/around one time, it is of utmost importance to verify if the preterm babies generate satisfactory immune response to all the vaccine components. Immunization of preterm infants from Poland and Spain with a 13-valent pneumococcal conjugate vaccine at the ages of 2, 3, 4 and 12 months led to lower but acceptable antibody levels (11). Based on the response to Haemophilus influenzae type b (Hib) vaccine, schedule modification (12) and need for boosters (13) were recommended in Japan and Poland, respectively. In Bangladesh, hepatitis B vaccine (Hep-B) generated protective, but, <100 mIU/ml anti-HBs titers in 26.4% Preterm infants (14). While revisiting data on immune response of preterm infants from different countries to hexavalent DTPa-HBV-IPV/Hib vaccine (>1600 preterm infants including 596 with GA ≤32 weeks, and 127 with GA <29 infants), excellent seropositivity against diphtheria (98.7%), tetanus (100%) and pertussis (92.4%) was recorded. However, for hepatitis B and Hib, seropositivity rates and antibody concentrations appeared to be lower (15). Based on the studies reported from Italy, it was surmised that though the administration of hexavalent vaccine leads to high seroprotection rates in preterm infants, lower antibody titers against HBV, Hib, poliovirus serotype 1, and pertussis) seem to have been associated with lower gestational age (16).

Under universal Immunization Program, Government of India offers a large number of free vaccines to its infants that are administered at recommended ages of the infants (17). To avoid multiple injections and enhance coverage, several combination vaccines are being introduced. India contributes to >3.5 million preterm births annually and thus protection of these infants against VPDs remains a top priority. Though immune response of full term (FT) infants is widely reported for the newly introduced vaccines (18–20), studies in preterm infants are restricted to BCG and hepatitis B (21–24). This study examines for the first time, antibody and cellular responses of PT infants to the pentavalent vaccine (Diphtheria and Tetanus Toxoids, *B. pertussis* (whole cell), hepatitis B and *Haemophilus influenza* Type b Conjugate) in comparison with their FT counterparts.

## Materials and Methods

### Sample Size

In the absence of data in preterm infants from India, the sample size for the study was calculated based on 10% inferiority (Sealed Enveloped Ltd. 2012. Power calculator for continuous outcome non-inferiority. Available from: https://www.sealedenvelope.com/power/continuous-noninferior/trial). At a significance level (alpha) of 5%, 90% power (1-beta), standard deviation of outcome = 2 and non-inferiority limit, d = 1, 69 samples were needed in each group. Considering 15% drop outs, 80 subjects/group were required.

### Study Population

**Figure 1:**
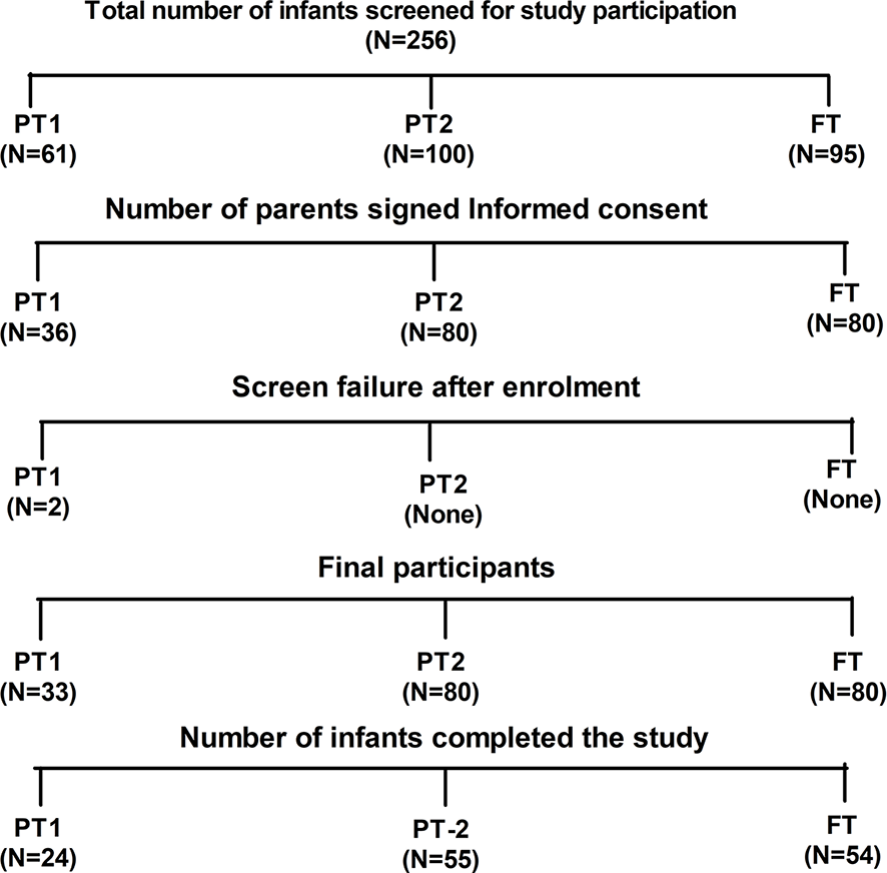
Study participant disposition. Out of 256 screened infants, parents of 193 infants gave informed consent for participation in the study. Two preterm infants from PT1 group were failed two meet inclusion criteria hence excluded from the study. Finally, 133 infants were able to complete the study.

The study was conducted during 2017 to 2018. **Figure 1** provides details of recruitment and follow up of study participants. After requesting 256 parents, 193 infants attending the pediatric department of Bharati hospital Pune for vaccination and routine checkup could be included. Among these, 33 were very preterm (gestation age [GA], 28–32 WK, PT1), 80 were moderate to late preterm (GA, 32–36 WK, PT2) and 80 were full term infants with >37 weeks of gestation (FT). All infants were born to HBsAg and HIV negative mothers. Exclusion criteria included past or current receipt of immunoglobulins, known suspected congenital or acquired immunodeficiency, chronic administration (defined as >14 days) of immunosuppressant or other Immune modifying drugs since birth (prednisone or equivalent for >0.5 mg/kg/day, inhaled or topical steroids were allowed). The study was approved by the institutional human ethics committee. After obtaining a written informed consent from the parents, demographic and baseline characteristics of the participants were recorded. HBV vaccine birth dose was given to the infants weighing ≥ than 2000 g. National immunization schedule for pentavalent vaccine was followed (17, 25). At the age of 6 weeks, first dose of pentavalent vaccine [DTwP-HBV-HIB vaccine, Biological E Ltd., India, (For details please refer supplementary information)**]** was administered. The subsequent doses of the same vaccine were given at 10th and 14th week of age. At the end of the follow up, blood samples could be collected from 54 (FT), 24 (PT1) and 55 (PT2) infants (**Figure 1**).

### Sample Collection and Processing

After written informed consent from parents, 1 to 3 ml venous blood (in majority, 1–1.5 ml) was collected from the infants in EDTA, at two time points. 1] Before the administration of first dose of pentavalent vaccine and, 2] One month after the last dose of pentavalent vaccine. For multiparameter flow cytometry analyses, whole blood was used within 3 h of collection and from remaining blood, PBMCs and plasma were separated by Ficoll hypaque density gradient method and cryopreserved at −196°C and −80°C, respectively, for recall response study and cytokine profiling.

### Multi-Parameter Flow Cytometry Analyses for Immune Cell Quantitation

**Figure 2:**
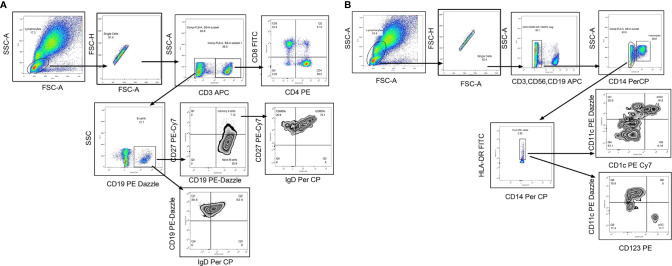
Flow cytometry gating strategy to identify lymphocyte subsets. Representative flowcytometry dot plots from the PBMCs of one of the study participants. **(A)** Gating strategy used to identify T cells and B cell subsets. The lymphocytes were live gated during acquisition using the side and forward scatter dot plot display. Lymphocyte population was further discriminated on the basis of CD3 expression. CD3 positive population was used to identify CD4 and CD8 T cells. Whereas CD3 negative population was used to identify B cells and its subsets such as memory B cells and class switched/unswitched B cells, class switched memory B cells (CSMB) and unswitched memory B cells (USMB). **(B)** Gating strategy used to identify antigen presenting cells (mDC, pDC and Monocytes).During acquisition, with the help of side and forward scatter, cells of lymphomonocyte gate were identified and the cells from this population were further distinguished on the basis of absence of lineage markers (CD3, CD19 and CD56). Lineage negative population was further discriminated as CD14+ and CD14- population to identify monocyte population. CD14 negative population was again distinguished on the basis of HLA-DR expression. Both positive (CD11c and CD1C) population from HLA-DR positive cells was identified as myeloid Dendritic Cells (mDC). Plasmacytoid dendritic cells (pDC) were identified as HLA-DR+CD11C−CD123 + cells. In infants with high NRBC (nucleated RBC) count, contour plot was used to identify lymphocyte gate for further analyses.

The whole blood was incubated with flurochrome conjugated antibodies (CD3, CD8, CD4, CD19, CD27, and IgD) in one tube and CD3, CD19, CD56, CD14, CD11C, CD1C, HLA- DR, and CD123 (please refer **Supplementary Table 1** for dye, manufacturer and clone of each antibody) in another tube for 30 min in dark at room temperature. After RBC lysis by 1× FACs lysis buffer (Cat no-349202 BD, Biosciences) and wash (FACS Buffer- PBS with 2% BSA), the stained cells were stored at 4°C in fixative (3% paraformaldehyde in PBS) till acquisition on Navios Flow cytometer (Beckman Coulter, Brea, Cal) using Navios acquisition software; acquisition was performed within 24 h of staining procedure. Gating strategy is depicted in **Figures 2A, B**. Total 100,000 events were recorded. After lymphocyte gate identification, lymphocyte population was segregated depending on CD3 expression and then CD3 positive cells were further discriminated on the basis of CD4 and CD8 expression to identify CD4T cells (CD3+CD4+) and CD8 T cell (CD3+CD4+) frequencies. CD3 negative population was separated on the basis of CD19 expression to identify B cell population (CD3−CD19+) that was categorized in memory B cells (CD3−CD19+CD27+) depending on their CD27 expression. To identify the class switched population, IgD based negative gating strategy was used. Class switched B cells were identified as CD3−CD19+IgD− and unswitched B cells as CD3−CD19+IgD+ whereas class switched memory B cells as CD3−CD19+CD27+IgD− and unswitched memory B cells as CD3−CD19+CD27+IgD+ cells (**Figure 2A**). The monocyte population (CD3−CD14+) was discriminated using CD14 expression on CD3 negative population. CD11c and CD1C expression on HLA-DR + CD3−CD56−CD19− cells was considered for detecting myeloid dendritic cell frequency whereas the plasmacytoid dendritic cells were identified as CD3−CD56−CD19− CD123+ HLA-DR +CD11 C− cells. The immune cell frequencies were expressed in proportion to total lymphocyte population (**Figure 2B**). The flow cytometry data were analyzed by using Flow Jo version7, Tree star.

### Plasma Cytokine Profiling by Cytometric Bead Array Analysis

Cytokine analysis was done using the Human CBA flex kits by simultaneous detection of IFN-γ, IL-2, IL-4, IL-5, IL-6, IL-10, IL-13, IL-12p70, IL-17, TNF-α, and IL-21 (BD Biosciences, San Jose, California). Employing thawed aliquots of plasma, CBA analysis was performed as per the manufacturer’s instructions. The samples were acquired on the same day on FACS Fusion machine by using FACS Diva software (version 3.0) FCAP Array Software (BD Biosciences, San Jose, CA, USA) was used to build standard curves and to calculate cytokine levels in plasma.

### Serology

The antibody (IgG) titers against all the components of pentavalent vaccine were determined by ELISA following instructions of the manufacturers. The anti-HBs levels were determined using TNO certified Anti-surase quantitative ELISA kits by General Biologicals Corporation, Taiwan. For determination of IgG antibody levels for Diphtheria, Tetanus, and *Haemophilus influenzae* b, EN ISO 9001:2000 certified kits from DeMeditec, Germany were used while for pertussis, kits from Euroimmune (Perkin Elmer company-EN ISO 9001, EN ISO 13485/CMDCAS) were used. The IgG antibody cut-off considered for protection was ≥0.01 IU/ml for diphtheria (26) and tetanus (27), ≥10 mIU/ml for hepatitis B (28) and ≥0.15 μg/ml for Hib (29). For long term protection against HiB, the cut-off of ≥1.0 μg/ml was considered. For pertussis, which has no recognized serological correlate of protection, IgG level >22 U/ml for whole cell, was considered as vaccine response (30).

### Recall Immune Response

The individual components of the pentavalent formulation were kindly provided by the Serum Institute of India, Pvt. Ltd. (SIIPL, Pune, India). Based on antibody titers against component antigens of pentavalent vaccine, ten representative samples including responders and non-responders against each of the antigen from all study groups (PT1, PT2 and FT) were selected (the details about antibody titers are provided in **Supplementary Table 2**). PBMCs of these samples obtained at post vaccination visits were revived and rested overnight at 37°C in 5% CO2 incubator. Next day, after live cell count by trypan blue, cells were washed with complete media (10% RPMI) and approximately 0.3 × 10^6^ cells were exposed to each of component antigens separately [(tetanus toxoid (3.3 LF/ml), diphtheria toxoid (2.4 LF/ml, PRP of Hib, 1 µg/ml), HBsAg (1 µg/ml) and the whole cell of pertussis (0.18 IOU/ml) for 6 h. A pre-stimulation period of 2 h was included before the addition of 10 µg/ml Brefeldin A(B7651; Sigma-Aldrich, St. Louis, USA) and anti-human CD107a (Degranulation marker) APC-Cy7. To check constitutive cellular frequency and their phenotypic and functional profile, cells without any stimulation were used as negative control. Anti-CD3 and anti-CD28 stimulation was used as positive control. After 6 h, the cells were kept overnight at 4°C followed by staining procedure as follows. Cells were washed with 1× PBS twice and stained by live/dead stain followed by surface staining for following markers. (CD3, CD4, CD8, CD45 RA, CD62L, CCR7, CD19, CD27, CD138, HLA-DR, CD11C, CD1C, CD80, CD86, CD300a, CD154, CD269, CD40, IgD, CD123, CD56, and CD14) Following fixation by 3% paraformaldehyde and thorough washing by FACS Buffer (1× PBS-EDTA with 2%BSA), cells were permeabilized by BD FACS Perm-II (Cat no-340973; BD Biosciences) and stained for intracellular markers (IFN-γ, TNF-α, and IL-2). Cells were acquired on FACS Fusion (BD, Biosciences). Approximately, lymphocyte gated 50,000 thousand events were acquired. Data were analyzed by Flow Jo tree star (version 7.6.5). Gating strategy for recall immune responses is denoted in **Supplementary Figure 1**. (**Supplementary Information** is provided for manufacturer, dye and clone of each antibody in **Supplementary Table 3**)

To assess the comparative efficacy of pentavalent vaccination in term and preterm infants, PBMCs exposed to short term antigenic stimuli with component pentavalent vaccine immunogens were analyzed for memory profile and functionality of B cells, T cells and dendritic cells. The memory profile comprised central (CD3+CD4+CD45RA-CD62L+CCR7+) and effector memory T cells (CD3+CD4+CD45RA−CD62L−CCR7−) and their ability to secrete cytokines such as IFN-γ, TNF-α and IL-2; CD107a expression was used as a marker for CD8 T cell-mediated cytotoxicity. In case of B cells, memory B cells (CD3−CD19+CD27+), plasma cell frequency (CD3−CD19+CD138+) and B cell maturation antigen expression (BCMA-CD269) density was determined. Myeloid (Lineage negative-HLA-DR+CD11C+CD1C+) and plasmacytoid DCs (Lineage negative-HLA-DR+CD11C-CD123+) were analyzed for up regulation of maturation markers such as CD80 and CD86.

The upsurge of 1.5 fold in cellular frequency and phenotypic and functional markers of immune cells after stimulation of respective antigens as compared to constitutive expression obtained in the PBMCs without any stimulation was considered as a positive response.

### Statistical Analysis

The data were analyzed using Graph pad prism (version 5). The nonparametric *Mann Whitey U test* was employed to compare the differences in immune cell subset frequencies and plasma cytokine levels between the groups. Student’s T test was used to compare antibody titers among the groups. Wilcoxon signed-rank test is employed to determine difference between immune cell subsets at baseline and after follow up visits. Spearman correlation is used to assess the bivariate correlation in between the parameters using SPSS. Multivariate and univariate analysis were performed using R programing.

## Results

Demographic details of 193 infants enrolled in the study are summarized in **Table 1**. Out of the total 193 infants recruited, 33 were very preterm [PT-1 (GA, 28–32 WK)], 80 were moderate preterm [PT-2(GA, 32–36 WK)] and 80 were full term [FT (GA, >37 WK)]. The study included 24, 55 and 54 follow up samples from PT1, PT2 and FT infant study groups, respectively. Overall, male gender was predominant in the preterm groups as compared to the term group. We did not observe any significant difference in age of infants at which pentavalent vaccination was administered. (One way ANOVA; at enrolment- p = 0.0807 and follow up visit- p=0.0984) (**Table 1**).

**Table 1:** Demographic characteristics of study groups.

Parameter	PT1 (28–32 weeks)	PT2 (32–36 weeks)	FT (>36 weeks)
Number recruited	33	80	80
Number followed up	24	55	54
Gestation age (weeks)	30.4	34.1	37.9
*Age at enrolment (mean weeks ± SD	10.2 ± 2.85	6.5 ± 1.94	6.4 ± 1.2
*Age at follow up (mean weeks ± SD	24 ± 1.4	20 ± 2.48	20 ± 2.5
M/F	22/11	64/16	36/44
Birth weight (kg) (mean weeks ± SD	1.02 ± 0.34	1.69 ± 0.43	2.9 ± 0.36

*Age difference among the groups was not significant (one-way ANOVA; at enrolment, 0.0807 and follow up visit, p = 0.0984)

Age at enrolment, before first dose of pentavalent vaccine.

Age at follow up, 1 month after last dose of pentavalent vaccine.

### Antibody Titers Prior to the First Dose of Pentavalent Vaccine (at 6 to 10 Weeks Age)

This time point reflects maternal transfer of antibodies (**Table 2**). In India, a birth dose is mandatory for hepatitis B and administration of this dose to preterm/term infants is deferred till the attainment of 2000g body weight. Therefore, immune response to hepatitis B component of pentavalent vaccine is considered separately.

**Table 2:** Pre and post pentavalent vaccination antibody titres against tetanus, diphtheria toxoid, *Haemophilus influenza* B and *Bordetella pertussis*.

Name of antibody	Baseline			Post pentavalent vaccination		
	PT1 (n=33)	PT2 (n=80)	FT (n=80)	PT1 (n=24)	PT2 (n=55)	FT (n=54)
**Anti-Tetanus- IgG**						
% Seroprotection and 95% CI (≥0.01 IU/ml)	100 (100–100)	100 (100–100)	100 (100–100)	100 (100–100)	100 (100–100)	100 (100–100)
% Seroprotection and 95% CI (≥0.1 IU/ml)	93.93 (89.91–100)	100 (100–100)	100 (100–100)	100 (100–100)	100 (100–100)	100 (100–100)
GMT	1.56	2.71	3.57	1.44	1.47	1.82
**Anti-Diphtheria-IgG**						
% Seroprotection and 95% CI (≥0.01 IU/ml)	87.87 (76–99.75)	92.5 (86.23–98.76)	97.5 (94.07–100.92)	100 (100–100)	100 (100–100)	100 (100–100)
% Seroprotection and 95% CI (≥0.1 IU/ml)	0	18.75 (9.47–28.02)	25 (15.51–34.48)	91.66 (81.6–100)	98.18 (95–100)	98.14 (95.19–100)
GMT	0.02	0.04	0.05	0.53	0.65	0.70
**Anti PRP-IgG**						
% Seroprotection and 95% CI (≥0.15 µg/ml)	81.81 (67.78–95.85)	70 (59.1–80.89)	72.5 (62.71–82.28)	95.83 (88.56–100)	100 (100–100)	98.14 (95.19–100)
% Seroprotection and 95% CI (≥1 µg/ml)	6.06 (0–14.74)	12.5 (4.63–20.36)	16.25 (8.16–24.33)	54.16 (36.03–72.3)	80 (70.49–89.5)	90.74 (84.38–97.09)
GMT	0.21	0.32	0.35	1.58	4.20	7.71
**Anti-Pertussis-IgG**						
% Seroprotection and 95% CI (>20 U/ml)	0 (0–0)	6.25 (0.49–12)	5 (0.22–9.77)	75 (59.23–90.76)	72.72 (62.14–83.31)	74.07 (64.47–83.67)
GMT	0.050	0.67	0.16	29.23	25.64	30.28

GMT, geometric mean titer; 95% CI, 95% confidence interval.

### Tetanus, Pertussis, Diphtheria, and HiB

In accordance with the mandatory immunization of pregnant women with tetanus toxoid during the third trimester, all the infants (except one from the PT-1 group) were circulative protective anti-tetanus antibodies (≥0.1 IU/ml) at the time of vaccination. In contrast, very few infants exhibited protective levels of anti-Pertussis toxin IgG antibodies (>22U/ml); none in PT1, 6.25% in PT2 and 5% in the FT groups suggestive of lack of these antibodies in the mothers. For diphtheria and HiB, respective antibodies were present in 87.9%, 92.5%, 97.5% and 81.81%, 70%, 72.5% infants in PT1, PT2 and FT categories, respectively. Of note, none of the infants from PT1 group circulated anti-Dtx-IgG antibodies at protective levels of ≥0.1 IU/ml while only 17.6% and 25% of the PT2 and FT infants exhibited such antibodies. For HiB, protective antibody positivity (1ug/ml) was recorded in 6.1% to 16.2% infants from the three infant groups.

**Figure 3:**
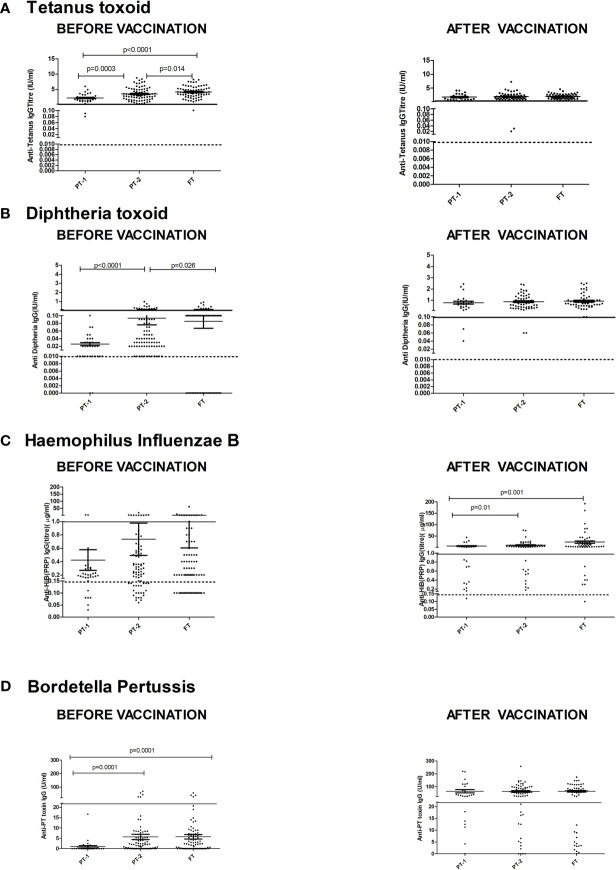
Antibody titers against component antigens of pentavalent vaccine. The vertical scatter dot plots present antibody titers against **(A)** Tetanus (anti-Ttx), **(B)** Diphtheria (anti-Dtx) toxoids, **(C)** anti-PRP (HiB), **(D)** anti-Pertussis toxin (*Bordetella*) before (at baseline) and after pentavalent vaccination. Dotted lines in the graph indicate seroprotective antibody titers. Error bars- Mean with SEM.

**Figure 3** depicts comparisons of antibody levels against the pentavalent antigens among the three study groups examined. The levels of anti-Ttx, anti-Dtx and anti-PT antibodies were significantly lower in PT1 than the PT2 infants (p = 0.0003, <0.0001 and <0.0001, respectively) while levels of anti-PRP antibodies were comparable (**Figure 3**).

### Antibody Titers 1 Month Post-Pentavalent Immunization (at 20 to 24 Weeks Age)

For tetanus, all the PT1/PT2 and FT infants exhibited anti-Ttx antibodies at the level of ≥0.1 IU/ml. Though 100% seroconversion was recorded for diphtheria in all the groups, lower antibody levels (<0.1IU/ml) were noted in 91.7% of PT1 infants as against comparable levels in PT2 (98.2%) and FT infants (98.1%). For HiB, 95.8% to 100% infants from different groups developed antibodies at ≥0.15 µg/ml level. However, almost half of the PT1 infants (54.2%) did not induce antibodies at ≥1 µg/ml as against 80 and 90.7% in PT2 and FT groups. For pertussis, 26% to 28% infants from all the groups lacked adequate antibodies at >22U/ml. Clearly, immune response to pertussis was suboptimal in all the immunized infants. Post-immunization anti-DTx, anti-TTx and anti-Pertussis (PT) antibody titers were comparable in all the groups examined. For HiB, the respective antibody titers were significantly lower in PT1 infants than in the PT2 and FT groups (p = 0.01–0.001) while no difference was noted among PT2 and FT infants (p >0.05) (**Table 2** and **Figure 3**).

Due to varied proportion of maternal antibody positives, we compared fold rise in antibody titers among maternal Ab positives and negatives in different study groups at 1 month post-immunization (**Table 3**). Immunization in the presence of high tittered maternal antibodies did not result in boosting effect as evidenced by post-immunization titers. For HiB, a significant proportion (31.8%, 68.2–75.9%) of Ab-positives did not induce desired antibody response while for pertussis and Hepatitis B, the response was independent of maternal antibody positivity. Whether such infants continue to circulate high antibody titers till receiving booster dose by 15th or 18th months of age in future remains an open question.

**Table 3:** Fold rise in antibody titres in presence and absence of maternal antibody (MT-Ab).

Immunogen	Infants with MT-Ab			Infants without MT-Ab		
	Number with ≥ 4fold rise in titers/Total Number with MT-Ab (%)			Number of infants seroconverting to protective levels/total number without MT-Ab (%)		
	PT1	PT2	FT	PT1	PT2	FT
Tetanus Toxoid	2/22 (9.1)	2/55 (3.6)	0/54	0/0	0/0	0/0
Diphtheria Toxoid	18/19 (94.7)	49/55 (89.1)	46/54 (85.2)	03/03 (100)	0/0	0/0
Hib (PRP)	15/22 (68.2)	41/55 (74.5)	41/54 (75.9)	0/0	0/0	0/0
Whole cell Pertussis	8/10 (80)	31/39 (79.5)	23/32 (71.9)	9/13 (69.2)	13/16 (81.2)	8/33 (24)
Hepatitis B surface antigen	12/13 (92.3)	36/39 (92.4)	33/36 (91.7)	9/9 (100)	11/14 (78.6)	36/36 (100)

Except for a small proportion of PT1 (2/22) and PT2 (2/55) infants, 4fold rise in anti-TT titers was not recorded post-immunization. It would be necessary to determine dynamics of these antibodies till boosted by an additional dose in 15th to 18th month.

MT-Ab, maternal antibody.

### Hepatitis B

#### Anti-HBs Levels Prior to Pentavalent Vaccine Administration

**Figure 4:**
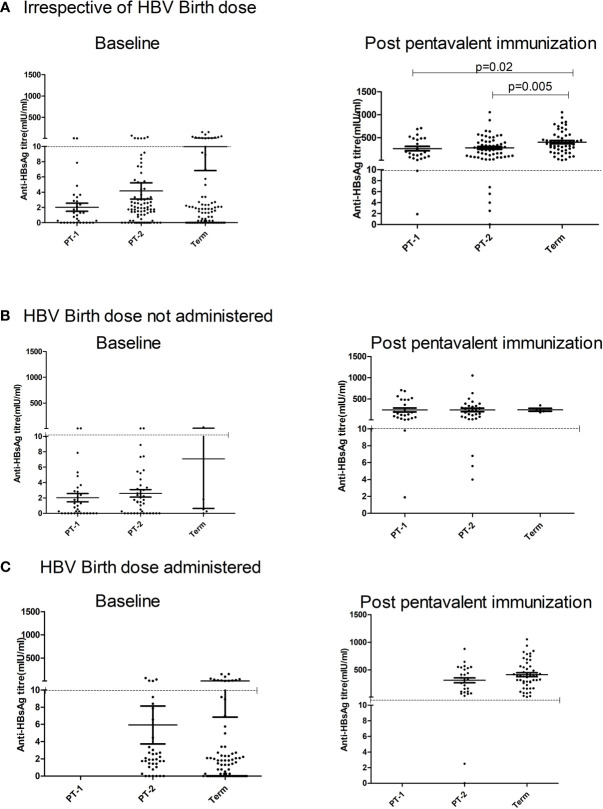
Effect of administration of Hepatitis B Vaccine dose at birth on anti-HBs titers at pre and post pentavalent vaccination. Hepatitis B vaccine birth dose is administered to infants weighing ≥ 2000 g. Pre (baseline) and post-pentavalent vaccination anti-HBs titers are depicted with respect to the administration of birth dose: **(A)** (Irrespective of birth dose), **(B)** (no birth dose administered) and **(C)** (birth dose administered). The vertical scatter dot plots in the figure represent the antibody titers against HBsAg at pre and post pentavalent visits. Dotted line in the graph indicates the seroprotective antibody titers. Error bars- Mean with SEM.

Maternal antibodies were detected in 57.7%, 70.7%, and 80% PT1, PT2 and FT infants prior to pentavalent immunization while 6.6% to 15% circulated protective anti-HBs levels. In PT2 infants receiving birth dose prior to pentavalent vaccine, 31/38 (81.57%) were anti-HBs positive while 30/42 (71.4%) without the birth dose circulated these antibodies (p>0.1). In FT and PT1 groups, 5/80 and 33/33 infants, respectively, did not receive the birth dose and hence such a comparison was not possible. Protective anti-HBs levels were independent of birth dose. Pre-immunization anti-HBs titers were comparable in all the groups with or without birth dose of the vaccine (**Table 4** and **Figure 4**).

**Table 4:** Anti-HBs IgG titres in all study groups categorized on the basis of HBV birth dose administration.

Parameter	Pre-pentavalent vaccination			Post-pentavalent vaccination		
Birth status (No)	PT1 (33)	PT2 (80)	FT (80)	PT1 (24)	PT2 (55)	FT (54)
	**Irrespective of birth dose**					
**Anti-HBs+ve**	19 (57.7%)	59 (73.7%)	55 (68.7%)	24(100%)	44 (97.8%)	54(100%)
**>10mIU/ml**	2(6.06%)	6 (7.5%)	12 (15%)	22 (91.7%)	40 (88.8%)	54(100%)
**GMT**	0.31	2.68	0.52	253.8	275.9	306.8
	**Birth dose administered**					
**Number**	**0**	**39**	**75**	**0**	**28**	**50**
**Anti-HBs+ve**	–	33(94.2%)	51 (68%)	–	27 (96.42%)	50(100%)
**>10 mIU/ml**	–	4 (10.25%)	11 (14.6%)	–	26(92.85%)	50(100%)
**GMT**	–	1.86	0.53	–	157.4	312.72
	**No birth dose**					
**Number**	**33**	**41**	**5**	**24**	**27**	**4**
**Anti-HBs+ve**	19 (57.7%)	29 (70.73%)	4 (80%)	22 (91.7%)	27 (100%)	4 (100%)
**>10mIU/ml**	2(6.06%)	2(4.87%)	1(20%)	22(91.7%)	24(88.88%)	4(100%)
**GMT**	0.31	2.14	1.09	122.4	130.98	306.76

GMT, geometric mean Titer. Values in bold represent total number of infants in the corresponding categories.

#### Anti-HBs Levels Post-Pentavalent Vaccine Immunization

One-month post-pentavalent immunization, 91.7% to 100% infants from all the three groups seroconverted to anti-HBs, 88.8% to 100% with protective antibody levels (GMT, 253.8–306.8). ~8% PT infants lacked protective levels of anti-HBs antibodies. Post-immunization anti-HBs titers did not vary significantly when the infant groups were compared with respect to the receipt of the birth dose (PT2 p = 0.45 and FT p = 0.15). Overall, FT infants developed higher antibody levels than the PT1 (p = 0.02) and PT2 (p = 0.005) infants (**Table 4**, **Figure 4**).

The serological results demonstrated clear differences in the immunogen-specific antibody positivity rates and proportions with protective titers in the infant groups examined. To understand basis for such differences, we examined circulating immune cells, cytokines and recall immune responses to immunogens of pentavalent vaccine.

### Enumeration of Circulating Immune Cells

The median frequency of immune cells (%) and the interquartile ranges (IQR) are summarized in **Table 5**.

**Table 5:** Lymphocyte proportions of Immune cells in all study groups at baseline and after pentavalent vaccination.

Type of immune cell	Enrolment			Post pentavalent vaccination		
	PT1 (N=33)	PT2 (N=80)	FT (N=80)	PT1 (N=24)	PT2 (N=55)	FT (N=54)
CD4% (median and IQR)	10.65 ± 5.97 (5.76–13.68)	12.94 ± 7.88 (5.94–17.93)	15.54 ± 7.88 (10.2–19.2)	12.19 ± 5.558 (8.54–15.85)	16.89 ± 7.7 (10.5–20.93)	18.99 ± 6.86 (12.9–25.43)
CD8% (median and IQR)	17.42 ± 10.27 (7.30–27.5)	10.86 ± 8.33 (4.84–13.6)	12.11 ± 7.14 (7.31–15.1)	19.98 ± 8.19 (16.95–24.38)	12.57 ± 6.07 (8.22–17.35)	15.49 ± 6.33 (11.65–18.35)
B cell % (median and IQR)	17.5 ± 9.93 (8.6–25.55)	16.73 ± 8.70 (8.95–23.5)	18.94 ± 10.23 (10.6–24.3)	18.23 ± 7.90 (11.15–25.95)	20.87 ± 8.83 (14.85–28.03)	22.63 ± 8.61 (15.83–27.45)
Class switched B cells % (median and IQR)	25.15 ± 14.53 (14.63–30.93)	25.73 ± 13.91 (14.8–32.9)	19.92 ± 11.95 (8.75–29.8)	28.48 ± 9.501 (20.7–35.1)	32.8 ± 15.38 (25.3–39.03)	29.82 ± 8.32 (24.73–34.38)
Unswitched B cell% (median and IQR)	73.07 ± 16.15 (63.35–82.95)	73.75 ± 14.37 (66.4–85.2)	79.86 ± 11.97 (70.2–91.25)	69.15 ± 9.824 (60.48–77.7)	65.88 ± 15.19 (60.5–73.23)	70.18 ± 8.32 (65.63–75.27)
Memory B cell% (median and IQR)	11.27 ± 15.17 (3.10–9.28)	9.86 ± 13.83 (3.63–8.63)	10.07 ± 10.33 (3.9–10.6)	13.98 ± 12.66 (6.063–15.08)	14.68 ± 16.57 (5.03–15.98)	8.03 ± 3.61 (5.17–10.3)
Class switched memory B cell % (median and IQR)	34.26 ± 22.91 (16.6–55.7)	32.42 ± 20.58 (14.6–44.9)	28.79 ± 17.97 (12.25–42.95)	38.37 ± 22.23 (18.05–52.7)	46.09 ± 22.26 (32.08–64.35)	49.79 ± 15.24 (38.68–61.2)
Unswitched memory B cells % (median and IQR)	64.17 ± 22.34 (43.75–79.98)	66.98 ± 20.37 (54.8–83.8)	71.21 ± 17.97 (57.05–87.75)	59.95 ± 20.82 (47.3–73.3)	53.01 ± 21.55 (35.65–67.58)	50.52 ± 14.74 (38.8–61.33)
Myeloid dendritic cells (mDC)% (median and IQR)	0.20 ± 0.12 (0.09–0.27)	0.35 ± 0.24 (0.19–0.47)	0.33 ± 0.22 (0.16–0.44)	0.30 ± 0.19 (0.17–0.47)	0.32 ± 0.19 (0.185–0.44)	0.32 ± 0.21 (0.18–0.44)
Plasmacytoid dendritic cells (pDC)% (median and IQR)	0.11 ± 0.09 (0.05–0.17)	0.2 ± 0.15 (0.09–0.26)	0.2 ± 0.14 (0.1–0.26)	0.11 ± 0.06 (0.05–0.17)	0.19 ± 0.19 (0.09–0.24)	0.15 ± 0.09 (0.08–0.19)
Monocytes % (median and IQR)	5.5 ± 3.82 (2.5–6.79)	7.75 ± 4.0 (4.77–10.4)	8.34 ± 4.24 (5.06–11.1)	7.4 ± 3.8 (5.033–10.63)	7.0 ± 3.4 (4.58–8.57)	6.26 ± 3.09 (3.58–9.28)

### Prior to Pentavalent Vaccine Administration

**Figure 5:**
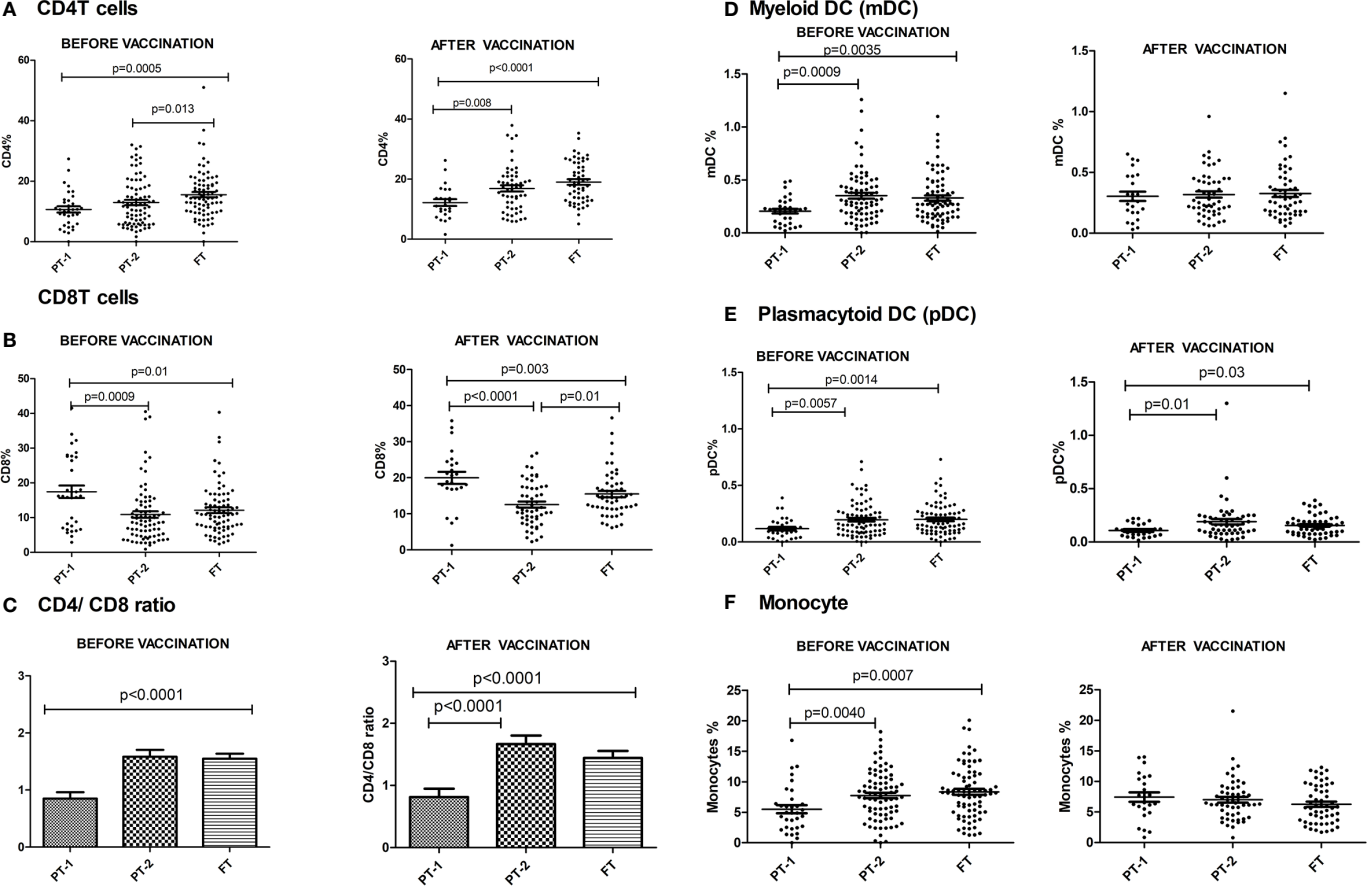
Frequency of major Immune cells in PT1, PT2 and FT infant groups prior to (at baseline) and one month post-pentavalent vaccination. The vertical scatter dot plots represent frequencies of different immune cells before and after pentavalent vaccination: **(A)** CD4 T cells, **(B)** CD8 T cells, **(C)** CD4/CD8 T cell ratio **(D)** Myeloid dendritic cells, **(E)** Plasmacytoid Dendritic Cells and **(F)** monocytes. Error bars- Mean with SEM.

The preterm infants showed lower frequency of CD4 cells (PT-1, 10.6%; PT2, 12.9%) when compared to the FT infants (15.5%, p <0.0001 and 0.008, respectively). The proportion of CD8 T cells was higher in PT1 infants (17.5%) than in the PT2 (10.86%, p <0.0001) and FT infants (12.11%; p = 0.01). CD4/CD8 ratio was equal to 1 or above in 33.3% PT1, 52.5% PT2 and 83.8% FT infants (**Figures 5A–C**). Additionally, PT1 infants were armed with lower number of antigen-presenting cells such as dendritic cells and monocytes. The proportions of both subsets of dendritic cells, myeloid dendritic cells and plasmacytoid dendritic cells were lower in the PT1 group than in PT2 (p = 0.0009 and 0.0057) and FT (p = 0.0035 and 0.0014) infants. Similarly, lower monocyte frequencies were recorded in the PT1 infants when compared to PT2 (p=0.004) and FT (0.0007) categories (**Figures 5D–F**).

### Post-Pentavalent Vaccination

At this time, the CD4 and CD8 frequencies were similar to enrolment i.e. lower CD4 and higher CD8 proportions in PT1 infants as compared to PT2 and term groups resulting in lower CD4/CD8 ratio in the PT1 infants. In contrast to pre-vaccination, the frequencies of monocytes and mDC at post-vaccination time point were comparable among all the three groups. However, pDC frequencies continued to be lower in the PT1 infants than in PT2 (p=0.01) and FT (p=0.03) infants.

### Plasma Cytokine Profiles Pre- and Post-Pentavalent Vaccination

**Figure 6:**
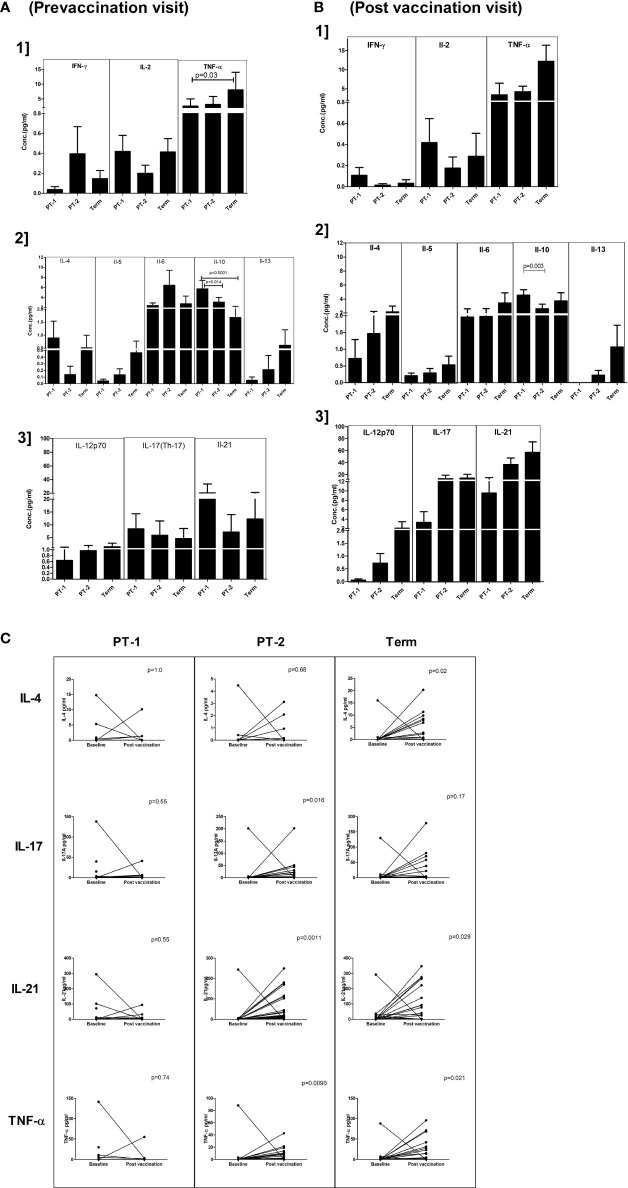
Plasma cytokine levels in PT1, PT2 and FT infants prior to **(A)** and post-**(B)** pentavalent vaccination. The vertical column bar graphs represent A1] Th1 cytokines (IFN-γ, IL-2, TNF-α), A2] Th2 cytokines (IL-4, IL-5, IL-6, IL-10, IL-13) and A3] Th17 and miscellaneous cytokines before vaccination; B1, B2, and B3 plots present respective cytokines at post-pentavalent vaccination. **(C)** The line graphs denote the changes in plasma IL-4, IL-17, IL-21, and TNF-α levels in all the infant groups before and after pentavalent vaccination. Error bars- Mean with SEM.

For this, 18, 36, and 33 paired serum samples, respectively, from PT1, PT2 and FT infants obtained pre and post pentavalent vaccination were tested. Among the Th-1 cytokines, TNF-α levels were significantly higher in term infants when compared to PT1 infants (p = 0.03) at enrolment visit. In contrast, the levels of IL-10, a Th-2 cytokine were higher in very preterm infants as compared to term infants (p = 0.001) and moderate preterm infants (p = 0.014) (**Figure 6**). Post-vaccination, IL-10 levels continued to be higher in very preterm infants than in the moderate preterm infants (**Figures 6A, B**). Marginal differences without any statistical significance were observed for the other cytokines tested at both the time points. With age, IL-21 and TNF-α level increased in the PT-2 and term infants while IL-4 increase was seen in the term infants (**Figure 6C**).

### Correlation Analyses

Next, we evaluated relationship of antibody titers against vaccine component antigens with variables such as gestational age and several immune cells/cytokines present in PT1/PT2/FT infants at the time of vaccination (**Table 6**). Of the immune cell parameters evaluated, univariate analysis revealed significant association of only few with antibody response. Gestation age was identified as a significant variable influencing antibody response to tetanus, diphtheria, pertussis and HBsAg, but not to HiB. For HiB and HBsAg, only single parameter was significant and hence multivariate analysis was not possible.

**Table 6:** Multivariate analysis to identify independent variables influencing antibody response to the pentavalent vaccine components*.

Immunogen	Variable	Univariate (p value)	Multivariate (p value)
**Tetanus toxoid**	Birth status	<0.001	0.0005
	CD4%	0.048	0.2
	CD8%	0.045	0.2
	pDC%	0.028	0.2
	Monocyte%	0.025	0.15
**Diphtheria toxoid**	Birth status	0.046	0.06
	CD4%	0.049	0.09
	Unswitched memory B cells%	0.042	0.16
	mDC%	0.002	0.2
	pDC%	0.007	0.03
	Monocyte%	0.04	0.6
***B. pertussis* (Whole cell)**	Birth status	0.049	0.054
	Monocyte%	0.01	0.055
	IL-2 (pg/ml)	0.02	0.055
	IL-6 (pg/ml)	<0.001	0.001
	IL-10 (pg/ml)	0.04	0.8
**HiB(PRP)**	mDC%	0.025	NA
**HBsAg**	Birth status	0.034	NA

*Parameters with significant influence on antibody titers in univariate analysis were included for multivariate analysis. NA, Not Applicable.

Anti-tetanus response was significantly associated with CD4, CD8, monocyte and pDC frequencies, but, multivariate analysis identified gestation age as the only predictor for antibody titers. In case of diphtheria toxoid, univariate analysis showed significant associations of humoral response with CD4, unswitched memory B cells, dendritic cells (mDC and pDC) and monocyte frequencies, however, multivariate analysis revealed a strong association with pDC only. For pertussis with compromised seroprotective titers in all the study groups, a significant association with gestation age, monocyte, and plasma levels of IL-6, IL-10, and IL-2 was found in univariate analysis, but plasma IL-6 levels emerged to be the significant parameter in multivariate analysis (**Table 6**).

### Recall Immune Responses to Constituent Immunogens of Pentavalent Vaccine

**Figure 7:**
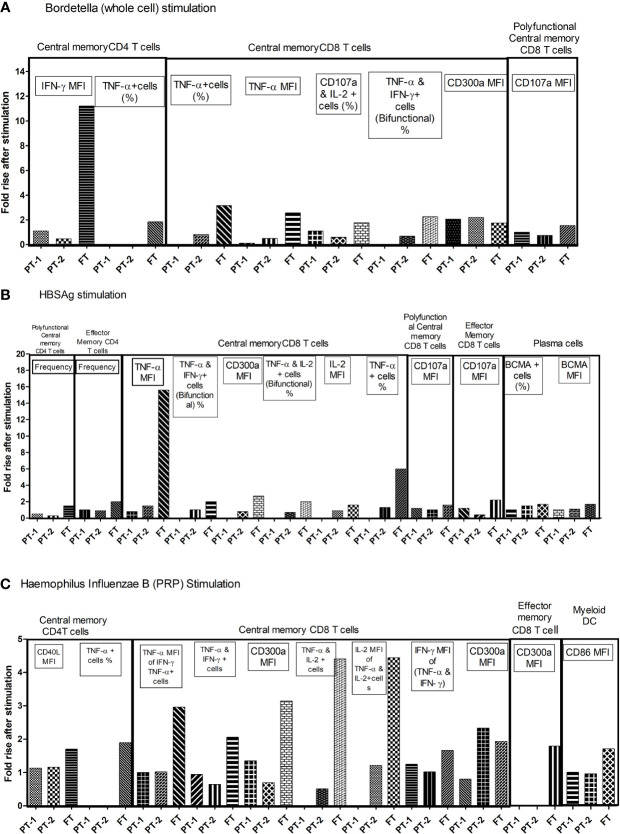
Fold changes in functional parameters of immune cells following individual pentavalent component antigenic stimulation of cultured PBMCs. Bar diagrams in the figure exhibit the fold rise in frequency of immune cells and expression of different markers after short term exposure to **(A)** Bordetella (Whole cell) **(B)** HBsAg **(C)** Haemophilus Influenzae B (PRP) in all study groups. The major memory T cell types are mentioned above the lines. Fold rise in relevant parameters are shown in boxes. In recall response analyses, PBMCs of infants without any stimulation were used as controls. The cellular frequency and their phenotypic and functional characteristics obtained in unstimulated PBMCs were considered as baseline values to estimate fold changes induced by stimulation with component antigens of pentavalent vaccine. [Concentration of antigens used for stimulation-(Tetanus toxoid (3.3 LF/ml), Diphtheria toxoid (2.4 LF/ml, PRP of Hib, 1 µg/ml), HBs Ag (1 µg/ml) and the whole cell of pertussis (0.18 IOU/ml); Incubation time, 6 h]. As against significant rise (>1.5 fold) of all the indicated markers in the FT group, PT1 documented 2.06 fold (Bordetella: central memory CD8 T cells, CD300a MFI) and 2.35 fold (HiB: TNF-α, and IFN-γ bifunctional central memory CD8 T cells). In PT2 infants, 2.2 fold (Bordetella: central memory CD8 T cells, CD300a MFI), 1.5 fold (HBsAg: central memory CD8 T cells, TNF-α MFI, and BCMA+ plasma cells) and 2.33 fold (HiB: central memory CD8 T cells, CD300a MFI).

To understand the comparative response of term and preterm infants to pathogen exposure, infant PBMCs from immunized infants were stimulated with component antigens of pentavalent vaccine. Live/dead staining of PBMCs demonstrated approximately 90% viability (median, 92.36; IQR, 85.47–96.32). Memory profile and functionality of B cells, T cells and dendritic cells was determined (**Figure 7**). None of the infants from all the study groups exhibited any modulation when the PBMCs were stimulated with tetanus or diphtheria. For the remaining three immunogens, birth status-specific alterations were noted. As against significant rise (>1.5-fold) of all the indicated markers in the FT group, the response of both PT1 and PT2 was much lower. PT1 documented an increase of 2.06 fold (*Bordetella*: central memory CD8 T cells, CD300a MFI) and 2.35-fold (HiB: TNF-α, and IFN-γ bifunctional central memory CD8 T cells). In the PT2 infants, central memory CD8T cells (CD300a MFI) increased 2.2 fold with pertussis and 1.02-fold, increased, central memory CD8 T cells (TNF-α MFI) and BCMA+ plasma cells raised 1.5-fold with HBsAg while central memory CD8 T cells (CD300a MFI) increased 2.33 fold for HiB. Overall, there was a prompt proliferation of central memory T cell compartment with Th1 cytokine secretion in term infants as compared to preterm infants. Immune cells of term infants showed highest capability to respond to the component immunogens, particularly inactivated whole cell of *Bordetella*, HBsAg and PRP moiety of *Hemophilus influenzae* B, suggestive of robust immunological memory development.

## Discussion

The primary aim of our study was to assess if the current national recommendations for pentavalent immunization of preterm infants are appropriate and ensure adequate immune response. For this, we first compared our results among FT infants with recent Indian studies (18). Post-pentavalent vaccination antibody positivity for tetanus, diphtheria, HiB and hepatitis B was similar to previous reports (18, 20, 24). In contrast, seroconversion rate against pertussis was alarmingly lower (74.1%). In view of the growing number of pertussis infections globally, this is indeed a matter of concern and additional studies are needed to assess if this is an isolated observation or a national concern. We would like to point out here that percent seroconversion to PT was found to vary from >95% (18, 20) to ~70% (31, 32). The vaccine administered during this study was provided by the government of India under the national universal immunization program (17).

Though we did not collect blood samples from the mothers, infant samples collected at ~6 weeks of age do reflect maternal antibody pattern in relation to the pentavalent antigens. Positivity rates were comparable to previously reported studies conducted during 2006 to 2009 (18, 20). Next, post-immunization antibody titers against the vaccine components were compared in relation to the gestational age. Different categories did not differ in mounting protective antibody response against tetanus and diphtheria. Though induction of anti-HiB antibodies at ≥0.15 ug/ml level was comparable in all the infants, the proportion of infants developing ≥1 ug/ml levels was lower preterm infants. Over 50% of the PT1 infants and 20% of the PT2 infants did not develop desired antibody levels and hence could be susceptible later. Of concern, suboptimal antibody response against pertussis was independent of gestational age (72–74%) and could be related to the vaccine response in general.

For hepatitis B, excellent and comparable (97.8–100%) anti-HBs positivity was observed in all the three groups, approximately 8% (PT1) and 11% (PT2) infants without protective anti-HBs levels. Nonetheless, FT infants with better developed immune system induced higher anti-HBs titers than the PT1 (p=0.02) and PT2 (p=0.005) infants. Administration of birth dose to the PT2 infants did not significantly influence antibody positivity/titers. Comparable anti-HBs response in full term, normal weight and underweight Indian infants was documented after receiving hepatitis B vaccine at birth and pentavalent vaccination as per recommended schedule (33). The data suggests that rather than birth weight, gestational age plays crucial role in deciding immune response.

Another important issue is the presence and titers of maternal antibodies in relation to birth status and effect on the development of antibody response post-vaccination. For tetanus, Diphtheria and HiB, almost all the infants were born to mothers with respective antibodies. For hepatitis B, rise in titers was independent of maternal antibody positivity (**Table 3**). In contrast, significantly lesser FT infants without maternal anti-pertussis antibodies (8/33, 24.2%) developed ≥4-fold higher titers than those with these antibodies (23/32, 71.9%, p<0.001). High anti-TT titers reflect mandatory immunization of pregnant women with TT. Except for a small proportion of PT1 (2/22) and PT2 (2/55) infants, 4 fold rise in anti-TT titers was not recorded post-immunization. Clearly, maternal antibodies did interfere with boosting effect. In fact, a drop was evident. It would be necessary to determine dynamics of these antibodies till receipt of the booster dose at 15 to 18 months age. Following a tetanus booster at 15 months, comparable humoral and cellular responses were recorded in FT and PT infants. At 15 months, premature infants exhibited lower levels of anti-tetanus antibodies, those born at a gestational age of < 32 weeks with lowest levels (34).

Taken together, our study revealed that as compared to the FT group Indian preterm infants develop adequate antibody response against tetanus and diphtheria, lower titers against HiB and HBsAg while suboptimal anti-pertussis response was independent of gestational age. To understand the basis for such differential responses, we determined relationship of gestation age with proportion of circulating immune cells and cytokines at pre- vaccination. Similar to an earlier observation, all the preterm infants exhibited lower frequency of CD4 and higher CD8 cells at the age of 6 to 8 weeks (35). Additionally, the PT1 infants were armed with lower number of antigen-presenting cells such as dendritic cells and monocytes. At this time, lower TNF-α and raised IL-10 levels characterized PT1 infants.

Three months later, PT1 infants continued to have lower CD4 cells and pDCs and, higher CD8 cells as well as IL-10 levels while the other parameters returned to levels similar to other infants. CD8 cells from cord blood of preterm infants were shown to be highly activated and hyper-responsive to inflammatory stimuli *in vitro* (36). Activation of T-cell–mediated immunity, particularly CD8 T cells, takes place during the first postnatal days in preterm infants with RDS (respiratory distress syndrome) and this activation is associated with development of bronchopulmonary dysplasia (BPD) (37). Pathogenesis of BPD is multifactorial (38). In the preterm infants, priming of adaptive immunity leading to augmented inflammatory response may be one of the factors contributing to the pathogenesis of BPD and other chronic complications. Indeed, history of RDS (Respiratory distress syndrome) in our infant series was gestational age-driven; as against 23.5% moderate preterm and 1.2% of FT infants, majority of the very preemies (70%) suffered from RDS (data not shown).

To identify parameters influencing immune response to individual components of pentavalent vaccine, multiple regression analysis was done. In univariate analysis, a significant association of GA with antibody response was found for all the antigens except HiB. Importantly, GA was identified as a significant independent variable influencing response to tetanus (p = 0.0005) and to a lesser degree for pertussis (p = 0.054) and diphtheria (p = 0.06). Though the response to tetanus was universal, GA did influence titers, increasing with GA.

Frequency of pDCs emerged to be the single independent variable positively impacting humoral response to diphtheria. It is pertinent to note that recombinant granulocyte-macrophage colony-stimulating factor (GM-CSF) could improve immune response to the diphtheria component in a multivalent vaccine (39). GM-CSF and IL-3 were able to efficiently promote pDC survival (40). A significant and direct correlation between the number of pDCs and the development of protective humoral immune response to measles vaccine at 3 months post-vaccination is noteworthy (41).

Pertussis component of the vaccine leading to suboptimal antibody response across all the infant groups is of special significance. In addition to IL-6 as the most significant independent factor (p=0.001), GA, monocytes and IL-2 seem to be impacting the antibody response as well (p = 0.054–0.055). Cytokine regulation and co-stimulatory molecules are pivotal to the immunological switch from innate to adaptive immunity. IL-6 was shown to contribute to the generation of *B. pertussis*-specific IL-17 responses (42). Studies have suggested that *B. pertussis* infection skews the host immune response toward the expansion of Th17 cells (43, 44). Further, IL-17 promoted macrophage killing of *B. pertussis* while depletion of IL-17 led to reduction in the efficacy of *B. pertussis* whole-cell vaccine (45). In mice, efficient clearance of *B. pertussis* and vaccine-induced immunity against the pathogen were shown to be IL-6 dependent (46). Despite identifying role of IL-6 in generating adequate anti-pertussis antibody response, the question of less efficacy of the vaccine even in term infants remains unanswered. It is indeed intriguing that despite lower frequency of CD4 and higher CD8 cells in the PT infants and lower number of antigen-presenting cells such as dendritic cells and monocytes in the PT1 infants, anti-pertussis antibody titers were comparable with the FT infants. Additional studies are needed to confirm this observation and to identify factors for the GA-independent immune response to pertussis. Whether BCG immunization selectively helped preterm infants in attaining anti-pertussis antibody titers similar to FT infants need to be examined.

We next attempted to evaluate if antibody negative infants will be able to mount cellular immunity responses if exposed to the respective pathogens. For this, recall responses were studied in 10 infants from each group. Data revealed that immunologic memory was not just a function of GA, but, varied with different immunogens. T cell or B cell subset proliferation was not seen for tetanus and diphtheria with high antibody response by all the infants and HiB with adequate protective antibodies.

Proliferation of central memory compartment of CD4 and CD8 T cells with polyfunctional response in FT infants with or without anti-pertussis antibodies revealed that these infants are likely to be protected following exposure to the pathogen. On the contrary, non-responders as well as responders from PT1 and PT2 groups were unable to develop immunological memory as indicated by the absence of proliferating memory T cells. Clearly, PT infants with immature immune system lacked efficient cellular response that could affect subsequent protection from *Bordetella*.

For hepatitis B component, the recall response was gestation age dependent and not influenced by the presence or absence of protective antibody levels. All FT infants mounted efficient antibody as we as cellular immunity in terms of memory CD8 T cells with TNF-α production. PT2 infants with or without protective antibody levels could induce similar responses. On the other hand, irrespective of the antibody status, very preemies were unable to induce rise in memory T cells. On exposure, these infants may be susceptible to the virus.

India being a TB-endemic country, BCG (live attenuated Bacille Calmette-Guérin) at birth is mandatory (17). BCG is a known immunomodulator and shown to reduce overall neonatal mortality as a non-specific beneficial effect (47, 48). In our study, all the FT infants received BCG at birth while the preterm infants were immunized with BCG after 34 weeks of gestation (34–20 weeks). Except for 4 infants receiving BCG and pentavalent vaccine simultaneously, the gap between these vaccinations was 1 to 4 weeks. In PT infants, proportions of mDCs, pDCs and monocytes before pentavalent vaccination were independent of timing of BCG administration (data not shown). Of note, in Gambia, administration of BCG to newborns at the time of priming markedly increased the cellular and Ab responses to the hepatitis B vaccine (at birth), but had only a limited influence on the cytokine response to tetanus toxoid and no effect on the Ab responses to tetanus and diphtheria toxoids (at 2 months age) (49).

In summary, our data provides comparative antibody response of Indian infants classified according to gestational age, to the component immunogens of the pentavalent vaccine. In view of the changing maternal antibody positivity and concentrations, such studies are essential at predefined intervals or whenever necessary. It is satisfying to note that irrespective of gestational age, all the preterm infants developed adequate antibody response against tetanus, diphtheria and, protective but lower antibody levels for HiB and hepatitis B as compared to their full-term counterparts. Suboptimal response to pertussis in all the infant groups emerged as a major concern. In addition to generating data on the relationship of circulating immune cells and cytokines with GA, the results revealed that both humoral and cellular immune responses of preterm infants were dependent on the type of the immunogen. Preterm infants born before 32 weeks of gestation may need an extra dose of pentavalent vaccine for long lived robust immune response. A further follow up till the receipt of booster dose is necessary to identify the window of susceptibility to these pathogens. For policy makers, our observations need to be extended to a large cohort representing different parts of the country.

## Data Availability Statement

The raw data supporting the conclusions of this article will be made available by the authors, without undue reservation.

## Ethics Statement

The studies involving human participants were reviewed and approved by the institutional human ethics committee, Bharati Vidyapeeth (Deemed to be) University, Pune. Written informed consent to participate in this study was provided by the participants’ legal guardian/next of kin.

## Author Contributions

VA conceptualized the study, reviewed results and analyses, and finalized the manuscript. AK-M planned and performed the work, analyzed the data, and wrote the manuscript. AM reviewed the results and contributed to the manuscript. NM, SP, and SL were responsible for subject recruitment, collection of relevant clinical information, and follow-up. AA performed parts of the experiments and provided help to AK-M. All authors contributed to the article and approved the submitted version.

## Funding

The study was funded by the Department of Health Research, Government of India under Grant in Aids Scheme Inter-Sectoral Convergence and Coordination for Promotion and Guidance on Health Research with the main objective on research studies with emphasis on public health (grant R.11012/03/2017-HR).

## Conflict of Interest

The authors declare that the research was conducted in the absence of any commercial or financial relationships that could be construed as a potential conflict of interest.

## Abbreviations

PT, Preterm; FT, Full-term; GA, Gestation age; VPD, Vaccine preventable diseases; CBA, Cytometric bead array; mDC, Myeloid dendritic cells; pDC, Plasmacytoid dendritic cells; BPD, Bronchopulmonary dysplasia ; RDS, Respiratory distress syndrome; GM-CSF, Granulocyte-macrophage colony-stimulating factor; BCG, Bacille Calmette-Guérin; GMT, Geometric mean titer; PRP, Polyribosylribitol phosphate; IQR, Interquartile range; MFI, Mean fluorescence intensity; RBC, Red blood cells; HiB, Haemophilus influenzae type B; NRBC, Nucleated RBC; HBsAg, Hepatitis B surface antigen; LF, Limits of flocculation; IOU, International opacity units; IU, International unit; PBMC, Peripheral blood mononuclear cells; BCMA, B cell maturation antigen.
