# Supplementary material for: Immune Response of Indian Preterm Infants to Pentavalent Vaccine Varies With Component Antigens and Gestational Age
Source: Front Immunol. 2021 Apr 23;12:592731. doi: 10.3389/fimmu.2021.592731 (PMC8102823; doi:10.3389/fimmu.2021.592731)
Supplement: Supplementary file 1 [file DataSheet_1.docx]

Supplementary Material

# Supplementary Information

1. **Pentavalent Vaccine details**

**Company – Biological E**

Each dose of 0.5 ml contains

- Diphtheria Toxoid ≤ 25 Lf (≥ 30 IU)
- Tetanus Toxoid ≥ 5.5 Lf (≥ 60 IU)
- B. pertussis (whole cell) ≤ 16 IOU (≥ 4.0 IU)
- rHBsAg 12.5µg
- Purified capsular

Polysaccharide (PRP) of

Hib Covalently linked to

20 to36.7 µgof Tetanus

Toxoid 11 µg

- Al+++ ≤ 1.25 mg
- Thiomersal 0.01% w/v

Further details can be accessed through following URL

<https://www.who.int/immunization_standards/vaccine_quality/pq_253_254_DTP_HepB_Hib_liquid_BiolE_PI.pdf>

1. **Specifications of flow cytometers used for study**
2. Immunophenotyping study-

Navios- Beckman coulter

Capacity-Two lasers, 8 parameters and six colors

1. Recall immune responses and Cytokine profiling study-

BD FACSAria™ Fusion - BD Biosciences

Capacity-Five lasers, 16 parameters and 14 colors

**Supplementary Table-1 Antibody details included in the immunophenotyping analyses of infant whole blood**

|  | **Antibody panel-1 Immunophenotyping** |  |  |  |  |
| --- | --- | --- | --- | --- | --- |
|  | **Antibody** | **Dye** | **Catalogue number** | **Company** | **Clone** |
| 1 | CD3 | APC | 344811 | BioLegend | Sk7 |
| 2 | CD8 | FITC | 344704 | BioLegend | SK1 |
| 3 | CD4 | PE | 344606 | BioLegend | SK3 |
| 4 | CD19 | PE Dazzle 594 | 302251 | BioLegend | HIB19 |
| 5 | CD27 | PE Cy7 | 356411 | BioLegend | M-T271 |
| 6 | IgD | Per cP Cy5.5 | 348233 | BioLegend | [IA6-2](https://www.biolegend.com/en-us/search-results?Clone=IA6-2) |
|  | **Antibody panel-2 Immunophenotyping** |  |  |  |  |
|  | **Antibody** | **Dye** | **Catalogue number** | **Company** | **Clone** |
| 1 | CD3 | APC | 344811 | BioLegend | Sk7 |
| 2 | CD19 | APC | 302212 | BioLegend | HIB19 |
| 3 | CD56 | APC | 318309 | BioLegend | HCD56 |
| 4 | CD14 | Per Cp Cy5.5 | 301823 | BioLegend | M5E2 |
| 5 | CD11c | PE Dazzle 594 | 337227 | BioLegend | Bu15 |
| 6 | CD1c | PE Cy7 | 331515 | BioLegend | L161 |
| 7 | HLA-DR | FITC | 327005 | BioLegend | LN3 |
| 8 | CD123 | PE | 306005 | BioLegend | 6H6 |

**Supplementary Table- 2 Details of the samples selected for recall immune responses post-stimulation with pentavalent vaccine immunogens**

| **Category** | **Sample-wise details of titres of antibodies against pentavalent antigens** | | | | | | | | | |
| --- | --- | --- | --- | --- | --- | --- | --- | --- | --- | --- |
|  | **1** | **2** | **3** | **4** | **5** | **6** | **7** | **8** | **9** | **10** |
| **PT1 (GA-28-32)** |  | | | | | | | | | |
| **Tetanus toxoid** | 0.73 | 0.86 | 2.89 | 0.81 | 1.99 | 2.90 | 3.50 | 2.45 | 2.98 | 4.11 |
| **Diphtheria toxoid** | 0.38 | 0.71 | 0.95 | 0.76 | 0.97 | 0.89 | 1.13 | 0.18 | 1.07 | 2.43 |
| **Whole cell Pertussis** | 4.16 | 11.30 | 17.88 | 67.51 | 33.05 | 38.96 | 23.06 | 215.17 | 218.86 | 119.21 |
| **Anti-HbS** | 9.79 | 141.63 | 488.78 | 1.91 | 708.83 | 365.33 | 65.99 | 16.48 | 443.02 | 483.11 |
| **HiB (PRP)** | 4.74 | 3.30 | 2.04 | 0.70 | 0.03 | 0.05 | 4.63 | 0.85 | 44.41 | 23.44 |
| **PT2 (GA-32-34)** |  | | | | | | | | | |
| **Tetanus toxoid** | 2.06 | 0.73 | 1.97 | 0.95 | 1.43 | 1.59 | 2.01 | 1.29 | 2.12 | 2.75 |
| **Diphtheria toxoid** | 0.30 | 1.10 | 0.25 | 0.35 | 0.27 | 0.12 | 0.99 | 0.48 | 0.26 | 0.06 |
| **Whole cell Pertussis** | 0.00 | 17.73 | 5.15 | 99.43 | 91.69 | 82.49 | 98.39 | 136.66 | 101.44 | 125.98 |
| **Anti-HbS** | 233.92 | 223.14 | 3.99 | 2.55 | 165.11 | 184.16 | 142.13 | 119.27 | 108.72 | 25.12 |
| **HiB (PRP)** | 24.50 | 1.10 | 2.82 | 73.47 | 4.99 | 1.57 | 75.36 | 0.63 | 4.94 | 5.87 |
| **FT(GA >37)** |  | | | | | | | | | |
| **Tetanus toxoid** | 1.23 | 1.75 | 3.00 | 3.87 | 2.82 | 1.30 | 1.50 | 1.85 | 2.64 | 1.02 |
| **Diphtheria toxoid** | 0.19 | 0.17 | 0.78 | 0.49 | 0.67 | 2.10 | 0.10 | 1.90 | 0.63 | 2.02 |
| **Whole cell Pertussis** | 8.90 | 12.16 | 5.00 | 9.32 | 34.90 | 58.01 | 74.97 | 63.41 | 117.79 | 116.81 |
| **Anti-HbS** | 186.79 | 167.58 | 635.60 | 13.17 | 522.31 | 116.81 | 292.14 | 213.00 | 687.24 | 371.61 |
| **HiB (PRP)** | 11.67 | 3.16 | 1.44 | 19.20 | 4.43 | 3.20 | 23.44 | 35.26 | 82.89 | 23.63 |

**Seroprotective titres-**

Tetanus Toxoid-0.1-01 IU/ml; Diphtheria Toxoid-0.01-0.1 IU/ml; Bordetella-20 IU/ML;

Anti-HBs-10 m IU/ml; HiB (PRP)- 0.15-1 ug/ml

**Supplementary Table-3 Antibody details included in the recall response analyses of infant PBMC**

| **Antibody panel-1 Recall responses (T cells)** | | |  |  |
| --- | --- | --- | --- | --- |
| **Anti-human Antibody** | **Dye** | **Catalogue number** | **Company** | **Clone** |
| Live Dead | APC | L3224 | Invitrogen |  |
| CD154 | Pacific Blue | 310820 | BioLegend | 24-31 |
| IL-2 | BV510 | 500337 | BioLegend | MQ1-17H12 |
| CD45RA | BV605 | 304133 | BioLegend | HI100 |
| CD197 | BV785 | 353229 | BioLegend | GO43H7 |
| CD300a | PE | a15778 | Thermofisher | MEM-260 |
| CD62L | PE-Dazzle | 304841 | BioLegend | DREG56 |
| CD16 | PE Cy5 | 302009 | BioLegend | 3g8 |
| IFN-γ | PE Cy7 | 506517 | BioLegend | B27 |
| CD107a | APC Cy7 | 328629 | BioLegend | H4A3 |
| CD8 | FITC | 344703 | BioLegend | SK1 |
| TNF-α | Per CP | 502923 | BioLegend | MAb11 |
| CD14 | BUV 395 | 740286 | BD Biosciences | M5E2 |
| CD4 | BUV 737 | 741823 | BD Biosciences | RPA-T4 |
| **Antibody panel-2 Recall responses (B cells)** | | |  |  |
| **Anti-human Antibody** | **Dye** | **Catalogue number** | **Company** | **Clone** |
| Live Dead | APC | L3224 | Invitrogen |  |
| CD19 | Pacific Blue | 302223 | BioLegend | HIB19 |
| CD138 | BV510 | 356517 | BioLegend | [MI15](https://www.biolegend.com/en-us/search-results?Clone=MI15) |
| HLA-DR | BV785 | 307641 | BioLegend | [L243](https://www.biolegend.com/en-us/search-results?Clone=L243) |
| CD123 | PE | 306005 | BioLegend | 6H6 |
| CD269 (BCMA) | PE-Dazzle | 357511 | BioLegend | 19F2 |
| CD38 | PE Cy7 | 356607 | BioLegend | HB-7 |
| CD11c | APC Cy7 | 337217 | BioLegend | [Bu15](https://www.biolegend.com/en-us/search-results?Clone=Bu15) |
| CD27 | FITC | 356403 | BioLegend | M-T271 |
| IgD | Per CP | 348233 | BioLegend | [IA6-2](https://www.biolegend.com/en-us/search-results?Clone=IA6-2) |
| CD40 | BUV395 | 565202 | BD Biosciences | 5C3 |
| CD80 | BUV737 | 751732 | BD Biosciences | 2D10.4 |
| CD1c | BV605 | 331537 | BioLegend | L161 |
| CD86 | PE Cy5 | 305407 | BioLegend | [IT2.2](https://www.biolegend.com/en-us/search-results?Clone=IT2.2) |
| **Lineage cocktail*** |  |  |  |  |
| CD3 | APC | 344811 | BioLegend | Sk7 |
| CD14 | APC | 325607 | BioLegend | HCD14 |
| CD56 | APC | 318309 | BioLegend | HCD56 |
| CD16 | APC | 302011 | BioLegend | 3g8 |
| ***Lineage cocktail Comprised CD3,CD14,CD56,CD16 antibodies (For Dendritic cell identification Besides these markers CD19 Positive cells were also excluded)** | | | | |

**Supplementary Figure-1**


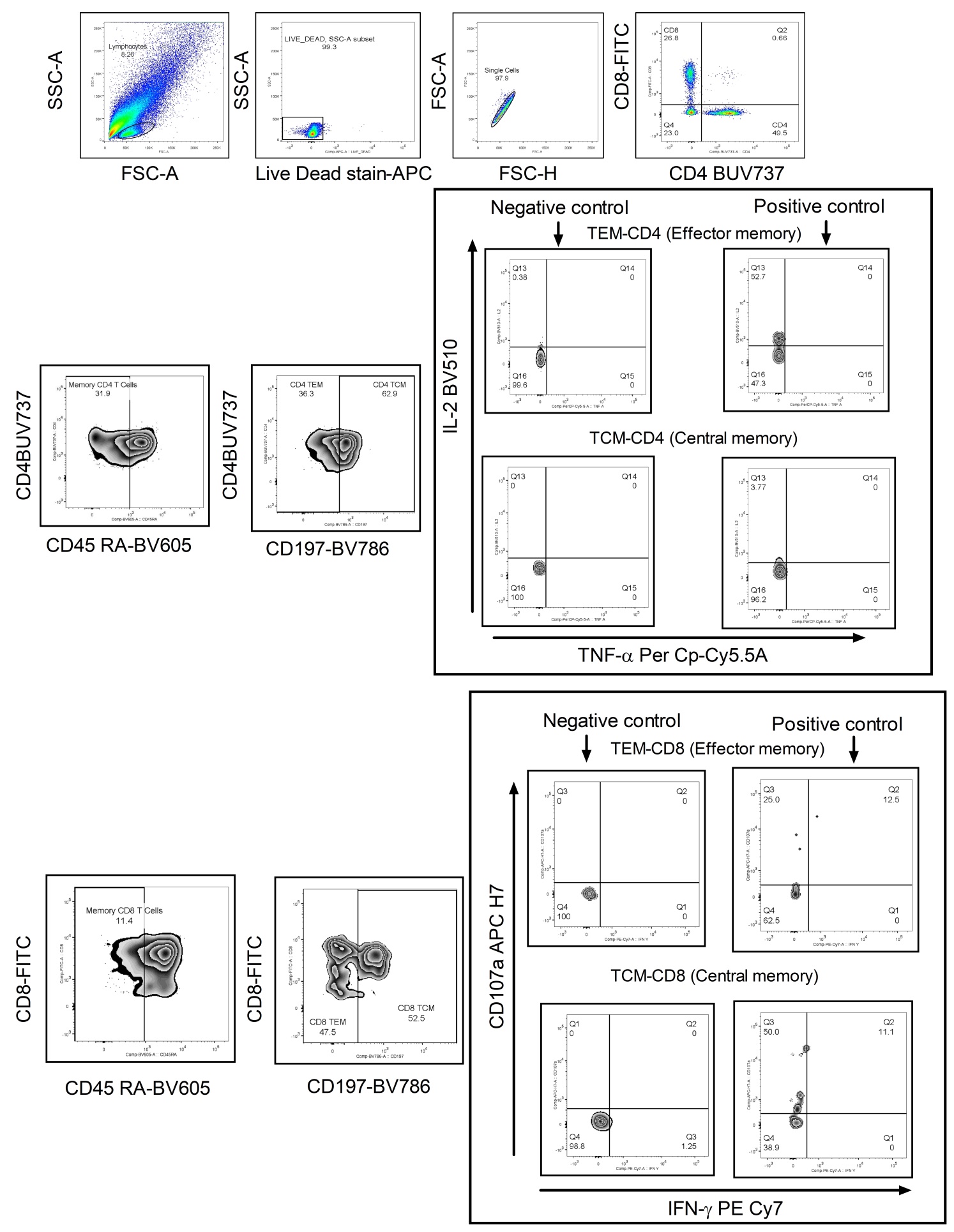


**Gating strategy used to identify memory T cells and subsets.** Representative flowcytometry dot plots from the recall responses analyses of one of the study participants. The lymphocytes were live gated during acquisition using the side and forward scatter dot plot display. Lymphocyte population was further discriminated on the basis of live dead stain and singlets. Further, on the basis of CD4 and CD8 expression, CD4 and CD8 T cells were identified. Memory T cells were identified on the basis of absence of CD45 RA expression. Memory T cells (CD4 and CD8 T cells) were further grouped as central memory (TCM-CD4/CD8) and effector memory (TEM-CD4/CD8) T cells. Then the memory T cell subsets were analysed for cytokine (IFN-γ,TNF-α & IL-2) secretion and CD107a expression. For CD4 memory T cell subsets representative dot plots for IL-2 and TNF-α are denoted. (Negative and positive controls) For CD8 memory T cell subsets representative dot plots for IFN-γ and CD107a are denoted. (negative and positive controls) In the similar way, all combinations of bifunctional (IL-2 &IFN-γ; TNF-α &IFN-γ; TNF-α &IL-2) and polyfunctional cells (IFN-γ,TNF-α IL-2 and CD107a) were identified for each memory T cell subset.
